# A retrospective cross sectional study assessing factors associated with retention and non-viral suppression among HIV positive FSWs receiving antiretroviral therapy from primary health care facilities in Kampala, Uganda

**DOI:** 10.1186/s12879-022-07614-w

**Published:** 2022-07-26

**Authors:** Lydia Atuhaire, Constance S. Shumba, Lovemore Mapahla, Peter S. Nyasulu

**Affiliations:** 1 Division of Epidemiology & Biostatistics, Faculty of Medicine and Health Sciences, Stellenbosch University, Cape Town, South Africa; 2 Makerere University/UVRI Infection and Immunity Research Training Programme, Entebbe, Uganda; 3 School of Nursing and Midwifery, Aga Khan University, Nairobi, Kenya; 4 Department of Population Health, Aga Khan University, Nairobi, Kenya; 5 School of Public Health, Faculty of Medicine and Health Sciences, University of the Witwatersrand, Johannesburg, South Africa

**Keywords:** ART, Retention, Lost to Follow up, Viral Load Suppression, Female Sex Workers

## Abstract

**Background:**

Patient retention in care and sustained viral load suppression are a cornerstone to improved health and quality of life, among people living with HIV. However, challenges of retention on ART remain among female sex workers (FSWs). We report lost to follow up (LTFU), viral load suppression, and the associated factors among FSWs that access HIV treatment at primary health care facilities in Kampala.

**Methods:**

We retrospectively abstracted and analysed patient management data of HIV positive FSWs who enrolled in care between January 2018 to December 2020. LTFU was defined as failure of a FSW to return for treatment at least 90 days from the date of their last clinic appointment. We defined viral suppression as having a last viral load of ≤ 1000 copies/ml preceding data abstraction. Data were analysed using Stata 15.1 software.

**Results:**

A total of 275 FSWs were included in our study sample. We found low retention of 85.1% (n = 234) at six months, corresponding to LTFU of 14.9 (n = 41) within the same period. Retention decreased with duration of being in care up to 73.5% (n = 202) at 24 months, and this translates to LTFU of 26.5% (n = 73). Viral load testing coverage was 62% (n = 132) and of these, 90.9% (n = 120) were virally suppressed. Factors associated with LTFU in univariable logistic regression; and viral load suppression in multivariable logistic regression models were; having a telephone contact (OR: 0.3, 95% CI: 0.1–0.9 p = 0.031), having enrolled in HIV care aged ≥ 35 years (OR: 0.5, 95% CI: 0.2–1.0 p = 0.048), (OR:0.03, 95%CI: 0.00–0.5, p = 0.016); and having good ART adherence (OR: 0.2, 95% CI: 0.1–0.5 p = 0.001), (OR:24.0, 95% CI: 3.7–153.4 p = 0.001) respectively. Having good ART adherence remained statistically significant (OR: 0.2, 95% CI: 0.08–0.53 p = 0.001) in multivariable logistic regression for LTFU.

**Conclusion:**

This study found low retention among HIV diagnosed FSWs in care. Viral load suppression was acceptable and comparable to that of the general population, however viral load coverage was low. Strategies that increase retention in care and access to viral load testing such as individual client centred care models are vital to improve retention and viral load coverage among FSWs.

## Introduction

At the end of 2019, key populations including female sex workers (FSWs) and their sexual partners accounted for 65% of new HIV infections globally [1]. Eastern and Southern Africa regions were mostly affected and in 2020 alone, key populations and their sexual partners accounted for 32% of new infections and the HIV prevalence was 30.6% [1]. The new Global AIDS Strategy accentuate commitment to achieving 95, 95, 95 new HIV cascade targets of testing, treatment, and viral suppression with emphasis on high-risk sub populations such as FSWs [2]. These targets are only achievable by increasing the reach and strengthening access to HIV services, reinforcing prevention interventions, expanding treatment, and ensuring retention on treatment to achieve viral load suppression for all HIV positive populations especially the high-risk groups such as FSWs.

The HIV epidemic in Uganda is generalised with an estimated HIV prevalence of 5.4% in 2020 among the population aged 15–49 [1]. Key Populations are most affected by the epidemic with FSWs having the highest HIV prevalence among all KP sub groups [1, 3]. According to Uganda AIDS Commission 2021 fact sheet, HIV prevalence is estimated at 31.3% among FSWs [4], a 4.6 times fold when compared to their female counterparts in the general population whose HIV prevalence is 6.8% [1]. Although Uganda has made substantial gains in HIV epidemic control and is one of the eight countries that achieved the 90-90-90, global HIV/AIDS 2020 targets [4], there are still population inequities in accessing HIV prevention and treatment services and FSWs are deeply affected. Indeed, the high prevalence among FSWs (31.3%), low ART coverage of 65% vs 96% among their female counterparts in the general population, low HIV status awareness of 88% vs 91% in the general population [1, 4] and sub-optimal condom use of 69.4% as well as active syphilis of 6.3% [1] in Uganda suggests that there are significant barriers for FSWs in obtaining access to a comprehensive package of essential health services. In Uganda sex work remains criminalised, leading to increased marginalisation and stigma [5]. Factors such as gender inequalities, physical violence, economic vulnerabilities, and discrimination hinder FSWs from accessing HIV prevention, treatment and affect FSWs’ retention in care thus suboptimal viral load suppression [6–8].

It is crucial to retain FSWs in HIV care and treatment programs to optimise viral suppression and improve health outcomes [9–11]. However, retention on ART and viral suppression among FSWs is a major challenge across different settings. A systematic review conducted among FSWs in Asia, Africa, North America, South America, and Central America and the Caribbean, reported a 38% current ART use among HIV infected FSWs with a 57% viral load suppression. The outcome estimates of ART use, were similar between high-, and low- and middle-income countries [12], implying that the challenge of continuity on ART is cross-cutting among FSWs regardless of the setting. Another systematic review conducted in sub-Saharan Africa (SSA), reported suboptimal continuity in care, despite expanded ART access among FSWs, with only 26–38% of HIV positive FSWs on ART reported by one of the studies reviewed [13, 14]. Similar retention and viral load suppression challenges among FSWs have been reported in a recent systematic review conducted in SSA (16). The review found that while retention and ART use may be high at the beginning of implementing retention interventions, the continuity in care is not sustained long-term or beyond the implementation period [15]. Indeed, results of a study conducted in Ivory Coast indicate steady loss of retention probability, reported at 75% at 6 months, 68% at 12 months, 55% at 24 months, and 47% at 36 months [16]. Similarly, a study conducted in Nigeria, reported an overall retention of 63.5%, 55.4%, 51.2%, and 46.7% at one year, two years, three years, and four years respectively, in a KP program where FSWs had a 54% majority representation [17].

Two studies have reported a combination of retention on ART and viral suppression among FSWs. The first study was conducted in Malawi and reported that, 69% of HIV-infected FSWs with a history of HIV care, only 52% reported current ART use, and of those, 45% were virally suppressed [18]. The second study was a cross-sectional respondent-driven sampling survey among FSWs in Zimbabwe and found that out of 67.7% of FSWs who reported ART use, 77.8% had HIV viral load < 1000 copies/ml, however among all HIV positive FSWs, 49.5% had a viral load < 1000 copies/ml [19]. Studies that have reported high retention and viral load suppression, are those conducted in settings with intensive follow up of participants while in HIV care. For example, an antiretroviral treatment adherence club intervention for FSWs implemented in Western Cape reported viral load suppression of < 1000 copies/ml among 20.5% of FSWs participants at baseline and the outcome increased to viral load suppression levels among 88.2% at 24 months of follow up [20]. Notably there are some studies that have reported non-significant differences in viral load suppression among FSWs even when they were conducted in settings that implemented intensive retention and ART use interventions. For example, Cowan [21] reported a viral load suppression of < 1000 copies/ml among 72% of FSWs in an intervention arm vs 67% FSW participants in a control arm at the end of follow up period in a FSW program in Zimbabwe where targeted combination prevention strategies were implemented.

In Uganda, multiple targeted HIV prevention and strategies for treatment continuity have been scaled up, however, challenges of retention and low viral suppression remain among FSWs [22]. Challenges of drop-out of care have been reported among women who engage in sex work as their main job [7, 23]. While the ART coverage among FSWs stands at 65%, their viral suppression rates are unknown [1] There is a dearth of information in Uganda about retention in care and viral suppression among FSWs, and to the best of our knowledge the only available results are from a respondent driven sampling study whose data was collected as far back as 2012 in Kampala [8]. The study reported very low performance, when measured against the 90–90-90 targets where, 45.5% of FSWs knew their HIV status, 37.8% were on ART and 35.2% were virally suppressed [8]. In this study, we report retention of HIV positive FSWs in HIV care, their viral load suppression rates, and the associated factors among FSWs that access HIV treatment at primary health care facilities in Kampala.

## Methodology

### Study design

This was a retrospective review of medical records of FSWs diagnosed with HIV infection. These were individuals enrolled into the HIV care program from January 2018 to December 2020 at the six-government primary health care facilities in Kampala, Uganda.

### Study setting

The study was conducted at six-government primary health care centres located in Kampala Uganda. These were Kawaala, Kisugu, Kiswa, Kitebi, Komamboga and Kisenyi, distributed across all the five administrative divisions of Kampala City, including Kampala Central Division, Nakawa Division, Makindye Division, Lubaga Division and Kawempe Division. The health centres serve as the main HIV outpatient clinics for the residential areas in Kampala and provide both curative and preventive health services that include HIV care for the general population and FSWs. The HIV services at the health centres are fully funded and implemented with support from the US Centres for Disease Control and Prevention.

### Study population

The study population was FSWs living with HIV who started ART between January 2018 and December 2020. There are over 8800 FSWs workers in Kampala [24] with HIV prevalence estimated at 31.3% among this population [4], which is 4.6 times higher than their female counterparts whose HIV prevalence is 6.8% [1]. We included all medical records of FSWs for review regardless of age at enrolment and as long as they were accessing HIV care at the government primary health care facilities in Kampala. FSWs medical records were excluded if they were missing data on key variables for the study including age, date of ART start, WHO staging and CD4 cell count at initiation. A total of 285 medical records within the study period were retrieved from Open MRS database and screened. However, only 275 records were included in the study and data abstracted, as the remaining ten had scanty information documented at enrolment.

### Study outcomes and variable measurement

The primary outcome of the study was ‘lost to follow up (LTFU)’ from HIV care, and a secondary outcome was virological non-suppression. LTFU from HIV care was defined ‘as failure of a FSW to return to the HIV clinic for ARV drug refill for at least 90 days preceding their last clinic appointment and not classified as transferred out to another clinic for treatment’. The definition of LTFU in this study was adopted from the Uganda Ministry of Health definition [25]. We assessed retention alongside LTFU, and participants were considered retained in care if they made a clinical visit within 90 days of a scheduled visit.

Virological non-suppression was defined as having a last viral load higher than 1,000 copies/ml, chosen based on WHO guidelines [10]. Viral load suppression was assessed for only those who had initiated ART at least 8 months before the date of data abstraction.

Following WHO strategic information guidelines [26], viral load testing coverage was assessed by calculating the proportion of FSWs on ART for at least 6 months with a current test result (results were deemed current if the next testing due date had not reached, that is, 12 months after ART initiation and every 12 months thereafter) [25].The exposure variables included age, marital status, level of education, year of ART initiation, presence of treatment supporter, having a telephone contact, WHO Stage, baseline CD4 count, if ever diagnosed with TB prior to the study, ART adherence status at the last visit, ever dropped out of care; returned for treatment continuity and if ART was initiated within ≥ 7 days following diagnosis of HIV/AIDS. Adherence was considered ‘good’ if patients reported no missed pills within three days prior to visiting the clinic for ART refill. For this study, every FSWs who had adherence documented as ‘good’ in their charts during the last visit prior to data collection was considered to have good adherence.

### Data collection

A data extraction form was developed to gather data of HIV FSWs routinely collected during case management. These are often reported to be associated with FSWs LTFU from HIV care. The following procedure were followed in the data collection process i) clinical data were extracted from HIV care/ART cards, a Ministry of Health medical chart for all ART patients used in all health facilities that provide ART services; ii) if CD4 counts and viral load results were not recorded in the HIV care/ART card, the laboratory records were reviewed; iii) data related to sex work were abstracted from key population specific registers that are routinely completed for all sex workers at enrolment or at any time a client identifies herself as a sex worker; iv) finally data were retrieved from the electronic medical record. The patient files were retrieved by a team of patient experts whose role at the facility is to retrieve charts for patients that routinely visit the clinics for drug refills. Assisted by one research assistant, data were collected by a member of the research team, who had taken lead in developing the protocol, study procedures, data collection and study ethics forms. The research assistant was a health worker familiar with HIV service tools and was oriented on the protocol, study procedures and how to extract data using a structured abstraction tool. Data collection took place between January and February 2021. To ensure quality of the data, all data abstraction forms were reviewed and 10% of the randomly selected FSW files were re-done by the research team. Data were entered into the Research Electronic Data Capture (REDCap) database.

### Data management

Data quality checks were done daily by crosschecking discrepancies, and completeness of data on all variables. The research team conducted real time form review and corrected missing or erroneous entries on site, where we could refer to the medical records. We checked for all forms of identifiable errors, and data completeness was conducted and exported into Stata version 15.0 for analysis.

### Data analysis

Data were analysed using Stata 15.1 software. The normally distributed continuous variables were described using mean and standard deviation, or else median and range where appropriate. The Shapiro Wilk test was used to assess for normality of continuous variables, and categorical variables were described as proportions and frequencies. A table was constructed to present data that describes the distribution of retention (dead, lost to follow-up and transferred out) at given time points (6, 12, 24 and > 24 months). Virological outcomes (viral load coverage and viral load suppression) were also presented in a table. Both univariate and multivariable logistic regression models were performed on both outcomes, lost to follow up and virological non-suppression. We used 0.2 level of significance to select variables to include in the multivariate analysis model. Stepwise logistic regression modelling was undertaken so that predictive variables for lost to follow up and non-virological suppression is carried out by an automatic process, while controlling for confounding effect of other covariates. All factors with p ≤ 0.05 were then considered statistically significant.

### Ethical considerations

The study was approved by the Uganda Virus Research Institute ethics committee and the Uganda national Council of science and technology (reference number HS-2665). We also obtained approval from Kampala Capital City Authority health office to allow us have access to patients’ data. To ensure confidentiality, no personal identifiers were abstracted, and participant unique identification numbers were used on data collection tools. The data collectors also signed a non-disclosure agreement.

## Results

### Baseline characteristics

In this study we extracted data for 285 FSWs enrolled in care between January 2018 to December 2020 and 275 FSWs were eligible for inclusion in the study **(**Table 1). At enrolment in care, half of the participants were aged 25–34 years 51% (n = 139) and a slightly higher number was either separated or widowed 38% (100). Approximately 48% (n = 131) had education level documented in their clinical records, of those 24% (n = 67) had attained primary education while 13% (n = 35) had no education and 11% (n = 29) had attained secondary education or higher. Majority of the participants were diagnosed with early stage of HIV disease at baseline with 82% (n = 223) categorised as having WHO stage 1 disease, 71% (n = 195) had baseline CD4 count of ≤ 500 (cells/μl) and 96% (264) had no signs of tuberculosis. Treatment initiation for the vast majority of participants followed the WHO recommendation of ‘test and start’ approach [10] with over 95% (n = 261) having initiated on ART within 7 days following HIV positive diagnosis. Details of the baseline participants characteristics are shown in Table 1

**Table 1:** Baseline characteristics of study participants at ART Initiation

Participant characteristics	Frequencies	Percentage
Health facility level		
HC III	209	76.0
HC IV	66	24.0
ART Initiation		
Within 7 days following diagnosis	261	96.0
After 7 days following diagnosis	11	4.0
Age at enrolment (years)		
< 25	92	33.4
25–34	139	50.6
> 35	44	16.0
Marital status at enrolment		
Never Married	78	29.5
Married/staying with partner	87	32.8
Widowed/Separated	100	37.7
Highest Education Level attained		
No education	35	12.7
Primary School	67	24.4
Secondary or higher	29	10.6
Unknown	144	52.4
Year of ART start		
2018	79	29.0
2019	108	39.7
2020	85	31.3
Has treatment supporter		
Yes	260	94.6
No	15	5.4
Has telephone contact		
Yes	259	94.2
No	16	5.8
Baseline WHO Stage		
WHO Stage I	223	81.7
WHO Stage II	40	14.7
WHO Stage III & IV	10	3.6
Baseline CD4 count (cells/μl) *1 person had missing baseline value*		
≤500	195	71.2
> 500	79	28.8
Diagnosed with TB during study period		
Yes	11	4.0
No	264	96.0
ART Adherence at the last visit		
1 = Poor	27	11.2
2 = Fair	10	4.2
3 = Good	204	84.6
Loss to follow up/drop out of care		
No	160	58.4
Yes	114	41.6
Reason for drop out of care		
Dropped out/LTFU	81	73.0
Dead	1	0.9
Transferred out	29	26.1
Client ever dropped and returned to Treatment for Tx Continuity		
No	240	87.3
Yes	35	12.7
Ever missed appointment for more than 3 days post clinical appointment		
No	95	34.7
Yes	179	65.3

### Lost to follow up and retention in HIV care

**Fig. 1:**
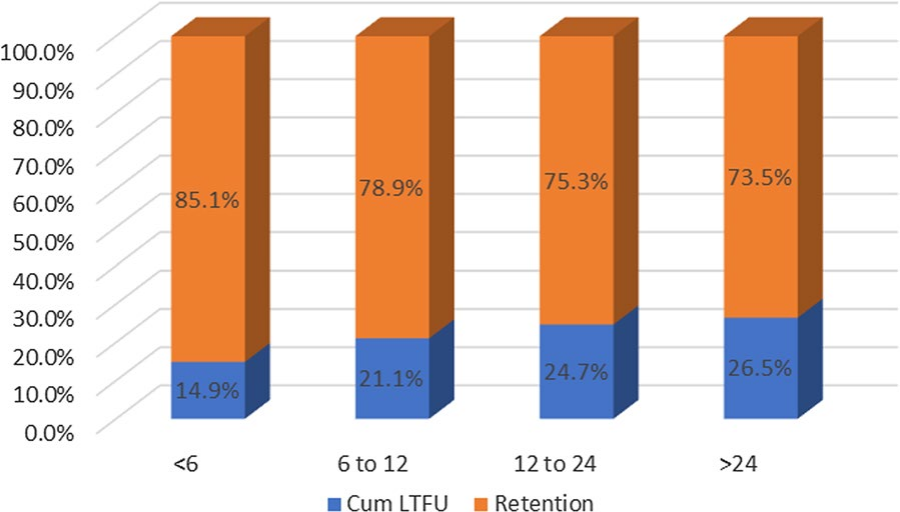
Lost to follow up vs retention (Months) at given time points among FSWs initiated on ART between Jan 2018–Dec 2020

As illustrated in Table 1, among 111 participants with reported reasons for drop out of care, 73% (n = 81) were lost to follow up, 26% (n = 29) had transferred out to seek care from other health facilities while 0.9% (n = 1) had died. Among 275 participants assessed for time-period to drop out of care, 14.9%, 21.1%, 24.7%, and 26.5% were lost to follow up at 6 months, 12 months, 24 months, and beyond 24 months on ART, respectively. These results demonstrate that higher losses from care happen within the first 6 months after enrolment in care. The overall retention was 85% at six months and it decreased to 74% at 24 months, implying reduced proportions of losses as participants stayed longer in ART care program. An illustration of the lost to follow up vs retention by time points is presented in Table 2 and Fig. 1.

**Table 2:** Retention and lost to follow up at given time points among FSWs enrolled in care between 2018–2020

Time point Factor n = 275	Cumulative attrition due LTFU only n (%)	Retention in care n (%)
< 6 months	41(14.9)	234 (85.1)
6 ≤ period ≤ 12 months	58 (21.1)	217 (78.9)
12 < period ≤ 24 months	68(24.7)	207 (75.3)
> 24 months	73(26.5)	202 (73.5)

### Virological testing coverage and non-viral load-suppression

Among the 275 participants assessed for virological status, 48% (n = 132) had tested and viral load test results recorded in their charts, 29.8% (n = 82) were due for testing but had not tested for viral load while 22.2% (n = 61) were not eligible for viral load testing as they had been in care for less than 6 months prior to the study. Viral load testing coverage among those eligible for viral load testing was 62% (n = 132). Of 132 participants with viral load results, 90.9% (n = 120) were virally suppressed with ≤ 1000 copies/mL **(Table ****3****).**

**Table 3:** Virological outcomes among participants enrolled on ART between Jan 2018–Dec 2020

Factor	N	%
Viral load testing coverage (n = 275)		
Tested and with viral load test result	132	48.0
Eligible but no viral load test done	82	29.8
Not eligible	61	22.2
Viral load suppression (n = 132)		
≤1000 copies/mL	120	90.9
> 1000 copies/mL	12	9.1

### Factors associated with lost to follow up

In univariate logistic regression analysis, the following factors were significantly associated with LTFU; age, marital status, having a telephone contact, and ART adherence at last visit. In the multivariable logistic regression ART adherence at last visit was independently associated with LTFU, Table 4.

**Table 4:** Logistic univariable and multivariable analysis of factors associated with Lost to follow-up

	Lost to follow up			
	Univariable analysis		Multivariable analysis	
Factors	OR (95% CIs)	P	OR (95% CIs)	P
Health facility level				
HC III	Ref.			
HC IV	1.0 (0.6–1.8)	0.990		
Days on ART after enrolment				
Within 7 days	Ref.			
After 7 days	1.2 (0.3–3.9)	0.797		
Age at enrolment in care				
< 25	Ref.		Ref.	
25–34	0.6 (0.4–1.1)	0.104	0.7 (0.4–1.2)	0.192
≥ 35	0.5 (0.2–1.0)	0.048	0.4 (0.2–1.1)	0.075
Marital status				
Never Married	Ref.			
Married	0.5 (0.2–0.9)	0.017		
Widowed/Separated	0.7 (0.4–1.3)	0.253		
Highest Education Level attained				
No education	Ref.			
Primary School	0.9 (0.4–2.1)	0.778		
Secondary or higher	0.4 (0.1–1.2)	0.098		
Has treatment supporter				
No	Ref.			
Yes	0.5 (0.2–1.3)	0.146		
Has telephone contact				
No	Ref.		Ref.	
Yes	0.3 (0.1–0.9)	0.031	0.3 (0.1–1.2)	0.086
Baseline WHO Stage				
WHO Stage I	Ref.			
WHO Stage II	0.7 (0.3–1.3)	0.261		
WHO Stage III & IV	0.1 (0.0–1.1)	0.062		
Baseline CD4 count (cells/μl)				
≤500	Ref.			
> 500	0.9 (0.5–1.6)	0.789		
Diagnosed with TB during study period				
No	Ref.			
Yes	0.3 (0.6–1.4)	0.128		
ART Adherence at the last visit				
1 = Poor	Ref.		Ref.	
2 = Fair	0.7 (0.1–3.0)	0.600	0.7 (0.2–3.5)	0.698
3 = Good	0.2 (0.1–0.5)	< 0.001	0.2 (0.1–0.5)	0.001
Ever missed appointment for more than 3 days post clinical appointment				
No	Ref.			
Yes	1.2(0.8–2.1)	0.392		

### Factors associated with virological suppression

In Table 5, the univariate logistic regression models for virological suppression, age, having a telephone contact, ART adherence at last visit, loss to follow-up, and reason for loss to follow-up were significant, at 20% level of significance. A multivariable logistic regression model showed that virological suppression was associated with having good ART adherence at last visit (OR:24.0, 95%CI: 3.7–153.4 P = 0.001) and having enrolled in HIV care aged ≥ 35 years (OR:0.03, 95%CI: 0.00–0.5, P = 0.016). There was significant (p-value = 0.001) 2300% increase in odds to viral suppression in the group which adhered to ART at last visit relative to those who had poor adherence to ART after adjusting for age, having a telephone contact and loss to follow-up. The ≥ 35-year age group at enrolment in care had an adjusted 0.03 times odds to viral suppression relative to the < 25-year age group.

**Table 5:** Logistic univariable and multivariable analysis of factors associated with virological suppression

	Virologically suppressed			
	Univariable analysis		Multivariable analysis	
Factors	OR (95% CIs)	p	OR (95% CIs)	p
Health facility level				
HC III	Ref.			
HC IV	3.3 (0.4–27.1)	0.257		
Days on ART after enrolment				
Within 7 days	Ref.			
After 7 days	0.3 (0.0–3.0)	0.294		
Age at enrolment in care				
< 25	Ref.		Ref.	
25–34	0.2 (0.0–1.7)	0.136	0.1 (0.0–1.3)	0.082
≥35	0.1 (0.0–0.8)	0.033	0.03 (0.0–0.5)	0.016
Marital status				
Never Married	Ref.			
Married	2.1 (0.5–9.3)	0.313		
Widowed/Separated	2.1 (0.5–9.3)	0.313		
Has telephone contact				
No	Ref.		Ref.	
Yes	7.8 (1.2–52.3)	0.034	6.5 (0.8–54.8)	0.087
Baseline WHO Stage				
WHO Stage I	Ref.			
WHO Stage II	2.2 (0.3–18.5)	0.451		
Baseline CD4 count (cells/μl)				
≤500	Ref.			
> 500	1.0 (0.3–3.7)	0.954		
ART Adherence at the last visit				
1 = Poor			Ref.	
2 = Fair	0.7 (0.0–14.0)	0.794	0.8 (0.0–39.5)	0.917
3 = Good	10.4 (2.4–45.5)	0.002	24.0 (3.7–153.4)	0.001
Loss to follow up/drop out of care				
No	Ref.		Ref	
Yes	0.4 (0.1–1.3)	0.129	0.5 (0.1–2.0)	0.306
Reason for drop out				
Dropped out/LTFU	Ref.			
Transferred out	0.1 (0.0–0.8)	0.034		
Drop out of care at given time points				
< 6 months	Ref.			
6 ≤ period ≤ 12 months	0.5 (0.1–3.4)	0.477		

## Discussion

This cross-sectional study assessed factors associated with retention and non-viral load suppression among HIV positive FSWs who were enrolled in care between January 2018 and December 2020 in primary health care facilities in Kampala, Uganda. The study found a high percentage of lost to follow up of 26% at 24 months of being in care. Retention in care was 85.5% at six months and it decreased to 73.5% at 24 months. Viral load testing coverage among those eligible for viral load testing was 62% (n = 132). The high LTFU and low viral load testing coverage may have been exacerbated by the COVID-19 lockdowns that led to disruptions of routine health care service delivery. Nonetheless, among the 132 participants with recent viral load testing results, 90.9% (n = 120) had viral load of ≤ 1000 copies/ml. This viral suppression rate is comparable to that of the general population of PLWHIV in Uganda [1, 4]. However, this observed viral load suppression is lower than the UNAIDS 95:95:95 target, although the trajectory is on the positive direction towards achieving the UNAIDS 95% viral suppression target by the year 2025 as projected [1].

The retention on ART of 85% at 6 months and 74% at 24 months, indicates early interruption in treatment among newly diagnosed FSWs. Retention on ART in Uganda is 98% in the general population (5) while retention in our study averages 80%, this is an 18% difference in retention observed among FSWs compared to the general population. This is a cause for concern as this is a high-risk group which has the propensity to facilitate transmission of HIV in the population. Previous studies have shown a similar pattern of high LTFU among FSWs [16, 17]. A retrospective cohort study in Côte d’Ivoire among FSWs found low levels of retention on ART of 75% after 6 months of initiation on ART and this dropped to 68% at 12 months [16]. Another retrospective cohort study among KPs in Nigeria showed a decline in care from 63.5% at ART initiation to 55.4%, after one year of follow up on ART [17]. Both studies showed a linear trajectory of decline in retention in care among FSWs consistent with what we observed in our study. Over the years interventions aiming at improving retention in care among FSWs have been scaled up, however findings from this study and many other studies show suboptimal effect to continuity on treatment [27, 28]. As such, adaptations in service delivery approaches are critical to realise improved outcomes including retention. Documented innovations and best practices point to service delivery approaches that are optimal, and the common theme is the need to identify individual challenges to continuity on treatment and use a case management approach to address the challenges [29]. It is also indicated that the differentiated models currently being implemented can be more successful if they’re all-encompassing to consider the client preferences and provision of broad package of services adapted to suit FSWs contexts [30]. Generally, retention efforts are successful when coordinated, implying that the FSWs, the health providers and the health system need to effectively play their part in facilitating efforts that are effective for promoting continuity in care. Specifically, efforts towards reinforcing continuity of treatment should happen early at treatment initiation so that individual challenges are identified, and a joint supportive plan to help the clients navigate the health system with ease is instituted.

Our study found 90.9% viral suppression among FSWs. This is comparable to the viral suppression of 90% among PLHIV in the general population in Uganda [4]. Although data are sparse on viral load suppression rate among FSWs in Uganda, our finding is consistent with the few available studies done in Uganda which show a high viral load suppression among KP cohorts including FSWs [31, 32]. However the results are contrary to studies conducted in Burkina Faso where the viral load was undetectable in 81.8% of FSWs [33]; in Tanzania study, the viral suppression was 50.6% after an 18 months of follow up of FSWs [34] and in Zimbabwe where the viral load was 72% [21]. The observed differences could be due to an exceptionally low viral load testing coverage, in that only 62% of FSWs eligible for viral load testing had tested for viral load and had documented viral load test result. In addition, we observed a relationship between ART adherence and viral load suppression, it is likely that patients who adhere to their clinical care visits, mostly follow viral load testing schedule and understand the benefits of adherence to ART treatment and as such, are self-motivated to seek a viral load test when they are due for testing.

The viral load coverage of 62% is extremely low, when compared to the 95% expected viral load testing coverage [25]. The challenges of low viral load testing coverage among FSWs are common in SSA countries [35] and the gap is not only seen among FSWs but also among other individuals in the general population. Currently the viral load testing coverage in Uganda is 85% [36]. Despite this, Covid-19 has most likely worsened the challenges of access to viral load testing. Considering that our study was conducted during the first year of COVID-19 pandemic, it is highly likely that the movement restrictions could have constrained patients from accessing sample collection centres for viral load testing. Furthermore, logistical challenges could have affected transportation of samples from the lower-level health facilities to the central testing laboratories due to the lock down regulations*.* Nevertheless, robust strategies must be instituted to improve access to viral load testing among FSWs. For example, community viral load sample collection has been recommended as a viable alternative that should be integrated into other community-based HIV services for FSWs and it has been successful in Zambia [37]. However, to ensure quality and accuracy of tests, HIV programs need to develop standard service guidelines for referencing during implementation of community based viral load sample collection. In addition, challenges with low viral load coverage could be addressed by enhancing efforts towards focused viral load uptake education aimed at disseminating information on benefits of routine viral load testing, relevance of the results, and clinical management.

Our study also found that younger FSWs aged below 25 years were likely to experience LTFU and be non-virally suppressed compared to the older FSWs. Our findings support the existing studies, although not conducted among FSWs, data shows that adolescent girls and young women living with HIV have lower uptake, delayed treatment initiation, and lower retention in care [38–40]. Furthermore, population-based surveys in SSA countries indicate that adolescent girls and young women living with HIV have lower rates of viral load suppression than women 25 years and older [41, 42]. Being an HIV positive young FSW adds up multi-layered issues related to negative social and economic challenges, increased gender based violence, stigma, rejection, inadequate social support and reduced educational opportunities [43, 44] which are all complex to manage. Targeted broader interventions have been recommended when designing HIV programs for young FSWs, and include parental and peer support, education, mental health and communication [45]. Other effective strategies to improve HIV service access include supporting KP-led community groups to engage in planning, implementation, and monitoring of FSW programs [11, 30]. Most critical, there needs to be intentional focus directed towards assessment of availability of HIV services tailored for the unique needs of young FSWs. Further, young FSWs need to be supported to meaningfully participate in decision making during the planning process to improving access of HIV services and continuity on treatment for young FSWs.

As seen in a study conducted in a rural district of Uganda [40] and in Kenya [39], we observed that FSWs with telephone contacts were less likely to be lost to follow up and virally non-suppressed than those without telephone contacts. In the recent past HIV programs are increasingly utilizing technology platforms to reach KPs in care [11]. Use of virtual communication methods and mHealth platforms are known to facilitate improved HIV service delivery [46], indeed our study clinics routinely send reminder text messages to clients before their clinic appointment days. Enhancing ‘Return to Treatment’ strategy is a high priority intervention suggested by WHO and Presidential Emergency Plan for AIDS Relief for all treatment sites [11, 35]. As such, partners at the helm of HIV programming in Uganda need to strengthen digital communication platforms such as automated text messaging and provision of rapid results by SMS. However, these interventions are effective when integrated within other broad strategies that support continuity in care. A comprehensive package of behavioural, biomedical, and structural strategies tailored to address individual needs and aligned to FSWs context will go a long way to improve continuity in care and viral load suppression.

In this study, FSWs whose ART adherence was categorised as ‘good’ at their last clinic visit had extremely high odds of a viral load suppression (2300%) compared to those whose adherence levels in their clinic records was documented as either ‘fair or poor’. Similarly, FSWs with good ART adherence had decreased odds to dropping out of care. Adherence to ART has widely been documented to be associated with viral load suppression [17, 21, 38]. FSWs are a known vulnerable group that continue to face barriers that make it harder to maintain regular clinical care and ART adherence [12, 28], continuation of intensive follow-up is required to support ART adherence for those in care and to bring back to care those who experience treatment interruption. To improve ART adherence, reduce treatment interruption and improve viral suppression among FSWs, diverse practices need to be adopted to facilitate continuation in care. Given the additional challenges brought by COVID-19, HIV programs are being encouraged to assess for FSWs contextual challenges to adherence and institute modifications to address both longstanding and COVID-19 associated challenges which compromise continuation on treatment [35]. Some of the interventions include building capacity of FSW-led organisations and empowering them to take lead in addressing numerous challenges that directly impact sustained adherence to treatment. Careful attention is needed in the first six months of enrolment when newly enrolled FSWs tend to drop out of care, such that a personalised adherence support plan is jointly agreed upon with FSWs and close ongoing monitoring is ensured.

This study had strengths and limitations; recruitment was done from all government primary health centres in Kampala city. This led to an increased representativeness of the sample for the study. The limitations in this study that should be considered while interpreting study findings include: (i) Utilization of secondary data, routinely collected for patient management. Such data sometimes have gaps and may not warrant rigor for scientific research. (ii) Data on key variable such as disclosure, education, mobility status and income status known to affect LTFU were missed; (iii) Uganda HIV programs do not have national patient unique identifiers, making it impossible to track patients who shift unannounced to get HIV care services from other facilities, this may have resulted in overestimation of FSWs counted as LTFU. However, there is a dedicated team of FSWs peers who follow up and document clients who don’t return for their drug refill and this might have possibly minimised wrong categorisation.

## Conclusion

This study has shown that the risk of lost to follow up among HIV diagnosed FSWs on HIV treatment is high and the occurrence mostly happen in the first six months after initiating ART. Interventions to improve retention such as intensive adherence support, immediate attachment to FSW peer supporters and provision of individual need-based care should target newly enrolled FSWs at ART initiation. Although viral load suppression was acceptable and comparable to that of the general population, viral load coverage was grossly low. To address challenges of low viral load coverage, strategies that increase access to viral load testing such as disseminating information on benefits of routine viral load testing, extending testing services nearer to the FSWs by conducting viral load sample collection from the communities will go a long way to improve viral load coverage among FSWs. Lastly, the observation of having telephone contacts and their association to reduce LTFU, calls for expansion of technological media advancements in HIV programming and leveraging on mHealth for patient follow ups for timely viral load testing and sustained retention into care for improved health outcomes among FSWs.

## Data Availability

The datasets generated and analyzed during the current study are not publicly available due to the conditions of ethical approvals of research among key populations but are available from the corresponding author on reasonable request.
